# Characteristics and burden of acute COVID-19 and long-COVID: Demographic, physical, mental health, and economic perspectives

**DOI:** 10.1371/journal.pone.0297207

**Published:** 2024-01-22

**Authors:** Manuel Leitner, Gloria Pötz, Martin Berger, Maria Fellner, Stephan Spat, Marisa Koini

**Affiliations:** 1 Division of Neurogeriatrics, Department of Neurology, Medical University of Graz, Graz, Austria; 2 digitAAL Life GmbH, Graz, Austria; Clinical Investigation Center, TUNISIA

## Abstract

**Background:**

COVID-19 infection and its associated consequence, known as long-COVID, lead to a significant burden on the global healthcare system and limitations in people’s personal and work lives. This study aims to provide further insight into the impact of acute and ongoing COVID-19 symptoms and investigates the role of patients’ gender and vaccination status.

**Methods:**

416 individuals (73.9% female) between the ages of 16 and 80 years (*M* = 44.18, *SD* = 12.90) with self-reported symptoms of long-COVID participated in an online survey conducted between March and May 2022.

**Results:**

6.0%, 74.3%, and 19.7% of all respondents reported having had an asymptomatic, mild, or severe acute illness, respectively. Out of all participants, 7.8% required hospitalization. The most prevalent symptoms during the acute infection (*Mdn* = 23.50 symptoms, *IQR* = 13–39) included fatigue, exhaustion, cough, brain fog, and memory problems. The median long-COVID disease duration was 12.10 months (*IQR* = 2.8–17.4). Among 64 inquired long-COVID symptoms (*Mdn* = 17.00 symptoms, *IQR* = 9–27), participants reported fatigue, exhaustion, memory problems, brain fog, and dyspnea as the most common ongoing symptoms, which were generally experienced as fluctuating and deteriorating after physical or cognitive activity. Common consequences of long-COVID included financial losses (40.5%), changes in the participants’ profession (41.0%), stress resistance (87.5%), sexual life (38.1%), and mood (72.1%), as well as breathing difficulties (41.3%), or an increased drug intake (e.g., medicine, alcohol; 44.6%). In addition, vaccinated individuals exhibited a shorter acute illness duration and an earlier onset of long-COVID symptoms. In general, women reported more long-COVID symptoms than men.

**Conclusion:**

Long-COVID represents a heterogeneous disease and impacts multiple life aspects of those affected. Tailored rehabilitation programs targeting the plurality of physical and mental symptoms are needed.

## Introduction

Since the World Health Organization (WHO) declared the coronavirus disease a global pandemic in March 2020, over 770 million confirmed cases [1] of COVID-19 have been recorded so far (11/2023). Although roughly 80% suffer a mild or moderate illness [2], some are at risk of a severe disease course, requiring intensive medical attention [3]. During the acute phase of an infection, symptoms typically encompass, but are not limited to, fever, cough, fatigue, dyspnea, or muscle aches [4, 5].

As the rate of infections remains elevated, the number of patients with persisting symptoms is increasing as well, leading to a significant amount of sickness rates and ongoing health challenges. Although there is a tremendous amount of heterogeneity in the definition of long-COVID in interventional studies [6], health experts defined the permanence of symptoms beyond four weeks after an initial infection subsided as long-COVID [7, 8]. Long-COVID includes both ongoing symptomatic COVID-19 (4–12 weeks) and the post-COVID-19 syndrome (+12 weeks) [9]. As persisting/ongoing symptoms such as cognitive impairment or fatigue can arise regardless of the initial illness severity [7, 10–12], a considerable number of individuals might develop long-COVID symptoms, especially if the virus continues to spread rapidly. These health issues could especially affect women and unvaccinated individuals, as research suggests a higher risk of long-COVID for women [7, 13, 14] and those without a COVID-19 vaccination [15]. Among various other risk factors, also a heightened body mass index [16] and a higher age [17] might increase the risk of developing long-COVID.

In general, the most common long-COVID symptoms include fatigue, chest pain, dyspnea, and cough [18], but also cognitive symptoms such as memory problems and brain fog are reported frequently [11]. As those symptoms may persist for months, the outbreak of the coronavirus disease has led to significant changes in the occupational [19] and personal lives of those affected. Individuals may need to consider reducing their working hours or are completely incapable of work [11, 20]. Others might be affected by psychological challenges, such as an increased level of depression, anxiety, anhedonia, or stress [21–23]. In addition, changes in peoples’ sex life were observed [7], such as a high prevalence of erectile dysfunction [24]. Finally, many also experience cognitive/mental limitations, such as global cognitive dysfunction [20], brain fog [25], attention disorders [22, 26] or memory problems [27].

Despite extensive examination of the diverse domains affected by individuals suffering from long-COVID, detailed information about the variety of symptoms and negative impact on work-life (e.g., changes in a person’s profession), private-life (e.g., stress, mood, need for assistance, and sex life), and cognition is scarce [11, 28].

Hence, there is an urgent need to investigate this complex medical condition and raise awareness regarding the ongoing effects of the COVID-19 pandemic. The current study aims at (a) describing the disease course, duration, and self-reported severity of participants’ acute COVID-19 infection, (b) characterizing the frequency and burden of acute and ongoing symptoms, (c) analyzing the impact of those symptoms on a variety of domains, including work life, financial losses, drug/medication intake, stress, mood, breathing, and sexuality, (d) investigating differences between vaccinated/unvaccinated individuals as well as between male and female respondents, (e) and analyzing the relationship between selected risk factors (e.g., BMI) and long-COVID symptoms.

## Materials and methods

### Participants and recruitment

This study represents a cross-sectional online survey available from March 29^th^ to May 3^rd^, 2022. All questions were presented in German. The study was approved by the ethics committee of the Medical University of Graz (34–166 ex 21/22). Informed consent was obtained by accepting an online data privacy statement, and participants were informed about the objective and duration of the study. All responses were anonymous and participants were free to withdraw from the study at any time, without providing reasons and without any negative consequences. Participants were recruited through social media advertisements, press releases, and information folders distributed in rehabilitation clinics. The online platform “LimeSurvey” was used to collect the data. ML, BM, GP and MK are specially trained in generating (online) questionnaires. Before and during the generation of the questionnaire, authors interviewed long-COVID patients, experts working with COVID patients (such as general doctors, neurologists, and nurse specialists), family members and the founder of a support group for long-COVID. Based on this information, we created the questionnaire used in this study and gave it to three long-COVID patients who evaluated it regarding its comprehensibility and goal-directedness. Afterwards, the questionnaire was adapted based on the feedback of the patients.

An a priori sample size calculation was conducted to determine the appropriate sample size for this study. The calculation was performed using the program G*Power (Version 3.1) and was based on an alpha level of 0.05, a statistical power of 0.80 and an anticipated medium effect size. The calculation indicated that a minimum of 159 individuals is required to detect the anticipated effects for all inferential statistical analyses.

The study sample comprised 416 individuals (73.9% female) between the ages of 16 and 80 years (*M* = 44.18, *SD* = 12.90) with a median active disease duration of 11–15 days (*IQR*: 6–10–16–20 days) and a median long-COVID disease duration of about 12 months (*IQR*: 2.77–17.36). As it was not mandatory to answer all questions provided, the sample size varies among questions. The majority reported being from Austria (91.1%), holding a university degree (26.3%), and reported working as an employee (70.2%). Detailed demographic information is provided in Table 1. Additionally, information regarding pre-existing health conditions was gathered and depicted in Table 2. There were no exclusion criteria in this study. However, the survey only targeted individuals with self-reported symptoms of long-COVID. Those with implausible values (e.g., year of birth “2022”) were excluded from the analyses.

**Table 1:** Demographic characteristics.

	*N* (total)	%	*M* ± *SD*	Range
**Total *n***	416			
**Age, years**	317		44.18 ± 12.90	16–80
**Gender**	333			
Female	246	73.9		
Male	87	26.1		
**Origin**	338			
Austria	308	91.1		
Germany	24	7.1		
Switzerland	4	1.2		
Netherlands	1	0.3		
France	1	0.3		
**Height, cm**	328		171.09 ± 9.22	123–200
**Weight, kg**	333		76.33 ± 21.41	43.0–178.0
**BMI, kg/m** ^**2**^	327		25.96 ± 6.56	15.4–62.5
**Education, years**	164		14.55 ± 4.12	6.5–25.0
**Education**	335			
Lower secondary school	2	0.6		
Compulsory schooling	9	2.7		
Apprenticeship	65	19.4		
High school diploma	79	23.6		
College	24	7.2		
University degree	88	26.3		
University of applied sciences	30	9.0		
Pedagogical university	16	4.8		
Others	22	6.6		
**Work**	363			
Employee	255	70.2		
Student	22	6.1		
Pupils	5	1.4		
Retiree	23	6.3		
Unemployed	7	1.9		
Self-employed	29	8.0		
Other	22	6.1		

**Table 2:** Pre-existing health conditions.

Pre-existing conditions (*n* = 217)	Frequency	Percentage (%)
Specification	151	
Dementia	0	0
Parkinson	0	0
Epilepsy	0	0
Multiple Sclerosis	0	0
Stroke	2	1.3
Hypertension	30	19.9
Diabetes	10	6.6
Chronic obstructive pulmonary disease	4	2.7
Cardiac insufficiency	5	3.3
Cancer	6	4.0
Other*	94	62.3

*Other pre-existing conditions include, for instance, allergies, asthma, depression, gastrointestinal disorders or hypo-/hyperthyroidism

### Procedure

The online survey assessed several domains, including demographic characteristics, pre-existing health conditions, duration of the initial COVID-19 illness, course of the disease, hospitalization rates, frequency and burden of acute (“which symptoms did you experience during the acute COVID-19 infection and how burdensome were these symptoms for you?”) and ongoing/long-COVID (“what long-COVID symptoms do you experience and how burdensome are these symptoms for you?”) symptoms (assessed utilizing a Likert Scale ranging from 1 to 5), the onset and relation between long-COVID symptoms, medical examinations, utilization of long-COVID therapies, changes in long-COVID symptoms, changes in breathing, stress, or mood, need for support, substance abuse, sexual alterations, financial changes and changes in the participants’ occupation due to long-COVID, information about the participants’ vaccination status and administered vaccines, training opportunities, and training motivation. Data was collected using single-choice and multiple-choice questions, which could be answered on a computer, mobile phone, or tablet. Participants had the option to provide additional information to specific questions by using text fields in the survey. The online survey took approximately 10 to 15 minutes to be completed.

### Statistical analysis

The data were analyzed using the statistics software SPSS (Version 29.0). We used descriptive statistics to describe the data. Chi-square or Fisher’s exact tests were computed to examine the relationship between categorical variables. Non-parametric tests (e.g., Mann-Whitney-U, Kruskal-Wallis) were applied to ordinal data or skewed continuous variables. Finally, univariate comparisons for continuous variables were performed using analyses of variance (ANOVAs) and corresponding post-hoc tests. We computed Spearman correlation coefficients to analyze relations between not normally distributed continuous or ordinal scaled variables, otherwise Pearson correlations were performed. A significance level of α = .05 was used. Effect sizes (e.g., Cohen’s *d*, η_p_^2^, φ_c_, *OR*, *r*) were calculated and specified in the corresponding analyses. All research data used in this study can be accessed in the S1 File (https://doi.org/10.3886/E196861V1).

## Results

### Acute COVID-19 infection: Symptoms and burden

**Fig 1:**
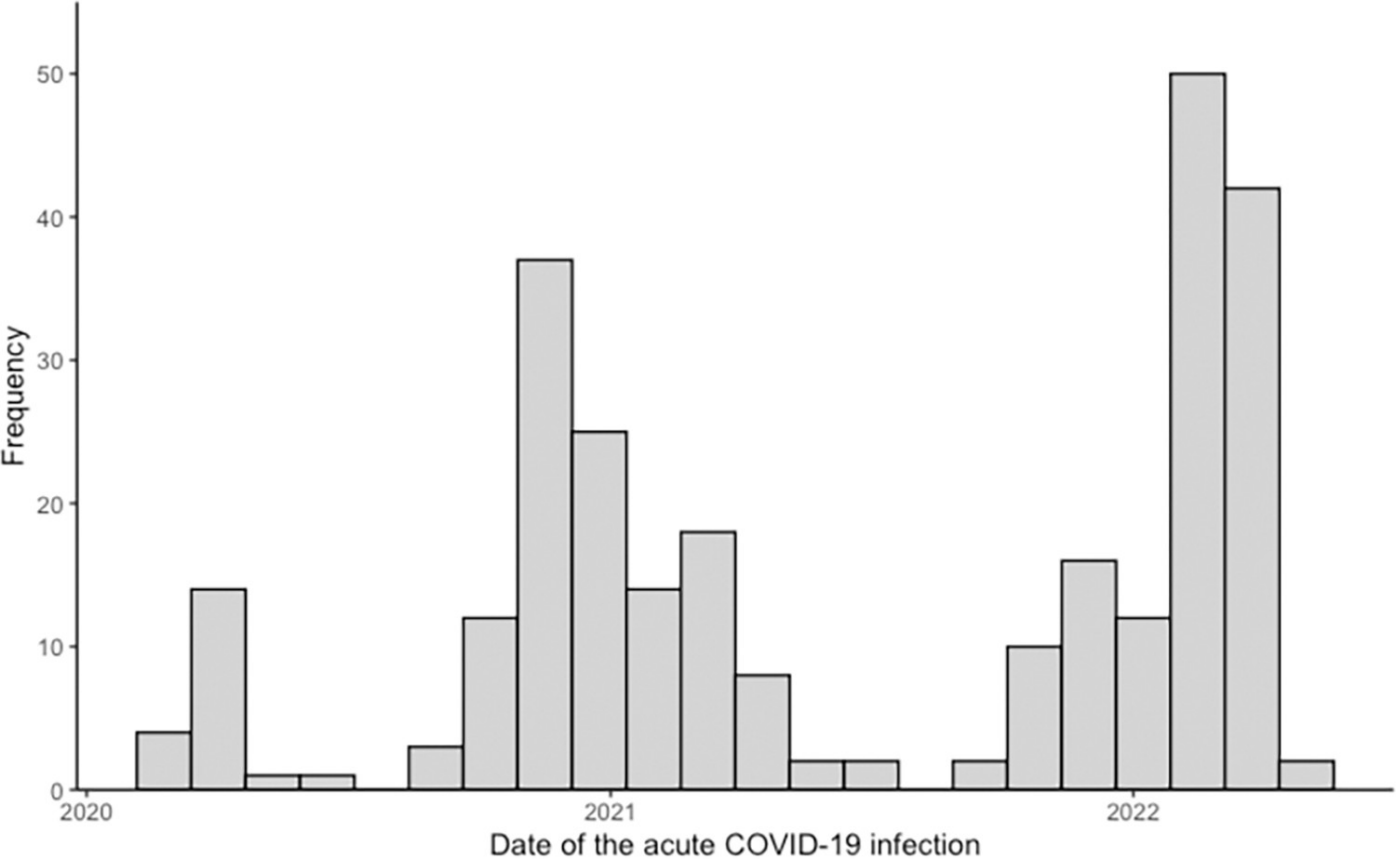
Infection frequency of survey respondents as a function of time.

**Fig 2:**
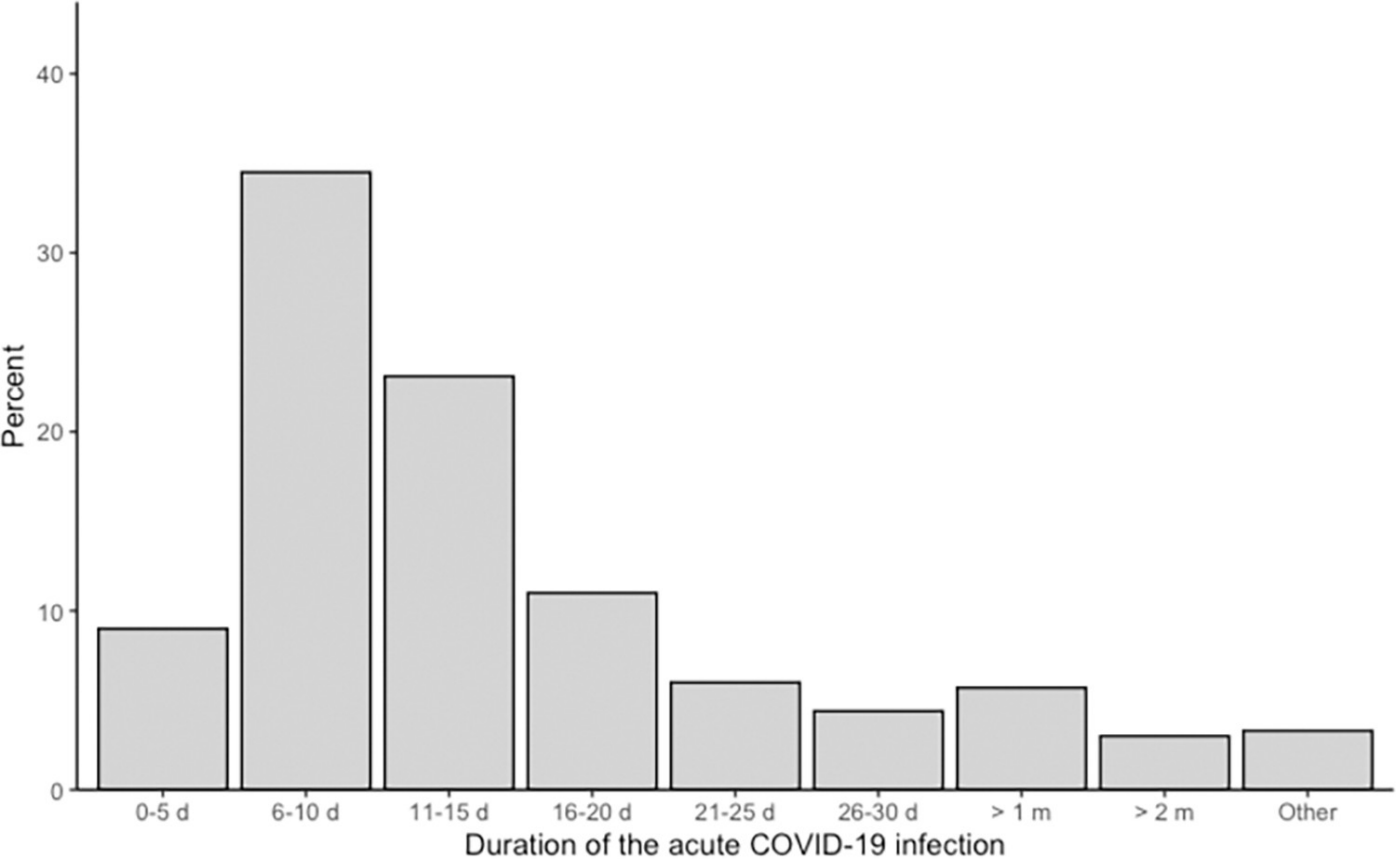
Duration of participants’ acute COVID-19 illness. *d* = days, *m* = months.

Individuals with self-reported symptoms of long-COVID and stating to have had a COVID-19 infection between February 2020 and May 2022 were included in the subsequent analyses. A graphic illustration of the infection frequency as a function of time is depicted in Fig 1. The duration of participants’ acute illness was positively skewed with a median of 11–15 days (*IQR*: 6–10–16–20 days) (Fig 2). Out of 299 respondents, the majority (74.3%) reported having had a mild course of disease (*n* = 222), while 6.0% (*n* = 18) had an asymptomatic or severe (19.7%, *n* = 59) disease course, respectively. Detailed information is presented in Table 3. Moreover, 7.8% stated they were admitted to the hospital. Of those dependent on hospital care due to the severity of their symptoms during the acute illness (*n* = 23), 60.9% were treated on a COVID-19 ward, while 17.4% were admitted to an ICU or did not further specify their hospital admission (21.7%). The median length of hospitalization was 0–10 days (*IQR*: 0–10–21–30 days) and only one respondent reported a hospitalization duration exceeding one month (Table 4).

**Table 3:** Detailed information about the duration and severity of participants’ acute infection.

Duration of acute infection (*n* = 299)	Frequency	Percentage (%)
0–5 days	27	9.0
6–10 days	103	34.5
11–15 days	69	23.1
16–20 days	33	11.0
21–25 days	18	6.0
26–30 days	13	4.4
> 1 month	17	5.7
> 2 months	9	3.0
Other*	10	3.3
**Disease severity** (*n* = 299)		
Asymptomatic	18	6.0
Mild	222	74.3
Severe	59	19.7

*Other information includes, for example, a still continuing infection

**Table 4:** Duration of hospitalization during the acute illness.

Hospitalization (*n* = 296)	*n*	Percentage (%)
Yes	23	7.8
No	273	92.2
**Duration of hospitalization** *		
0–10 days	12	66.7
11–20 days	2	11.1
21–30 days	3	16.7
31–60 days	0	0.0
> 2 months	1	5.5

*Duration is given for those hospitalized and providing further information (*n* = 18)

**Fig 3:**
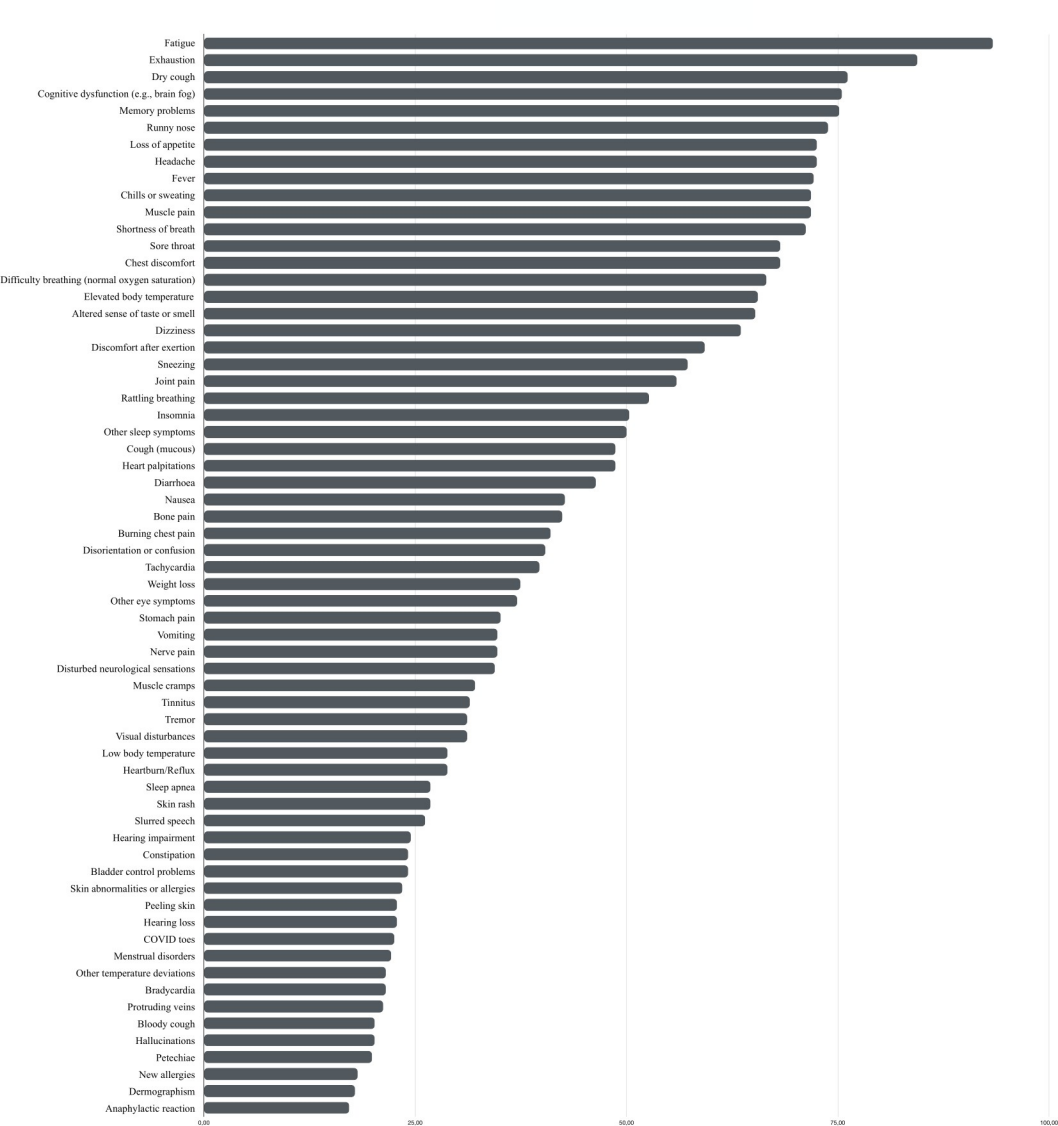
Percent of COVID-19 symptoms during the acute phase of the illness.

The participants experienced a median number of 23.5 different symptoms (*IQR*: 13–39 symptoms) during the acute phase of their illness. The most common initial symptoms included fatigue (93.4%), tiredness/exhaustion (84.4%), dry cough (76.2%), cognitive dysfunction (e.g., brain fog; 75.5%), poor memory (75.2%), a runny nose (73.8%), headache or headache-associated symptoms (72.5%), loss of appetite (72.5%), fever (72.2%), sweating or chills (71.9%), muscle aches (71.9%), and dyspnea (71.2%). A comprehensive list of symptoms can be found in Fig 3 and S1 Table.

The subjective burden of those symptoms during the acute and ongoing phase was assessed using a 5-point Likert Scale (*1* = very mild to *5* = very strong). Exhaustion (*M* = 4.13, *SD* = 0.94), an altered sense of taste and smell (*M* = 3.97, *SD* = 1.31), fatigue (*M* = 3.96, *SD* = 1.02), discomfort after physical exertion (*M* = 3.82, *SD* = 1.04), headache or headache-associated symptoms (*M* = 3.76, *SD* = 1.18), and dyspnea (*M* = 3.59, *SD* = 1.22) were reported to be the most significant burdens during the acute illness (S1 Table). In general, respondents experienced a very mild (26.4%) or mild (15.4%) burden caused by their symptoms, while the majority reported a moderate (22.1%), strong (20.0%), or very strong (16.1%) burden, respectively.

### Long-COVID: Symptoms and burden

**Fig 4:**
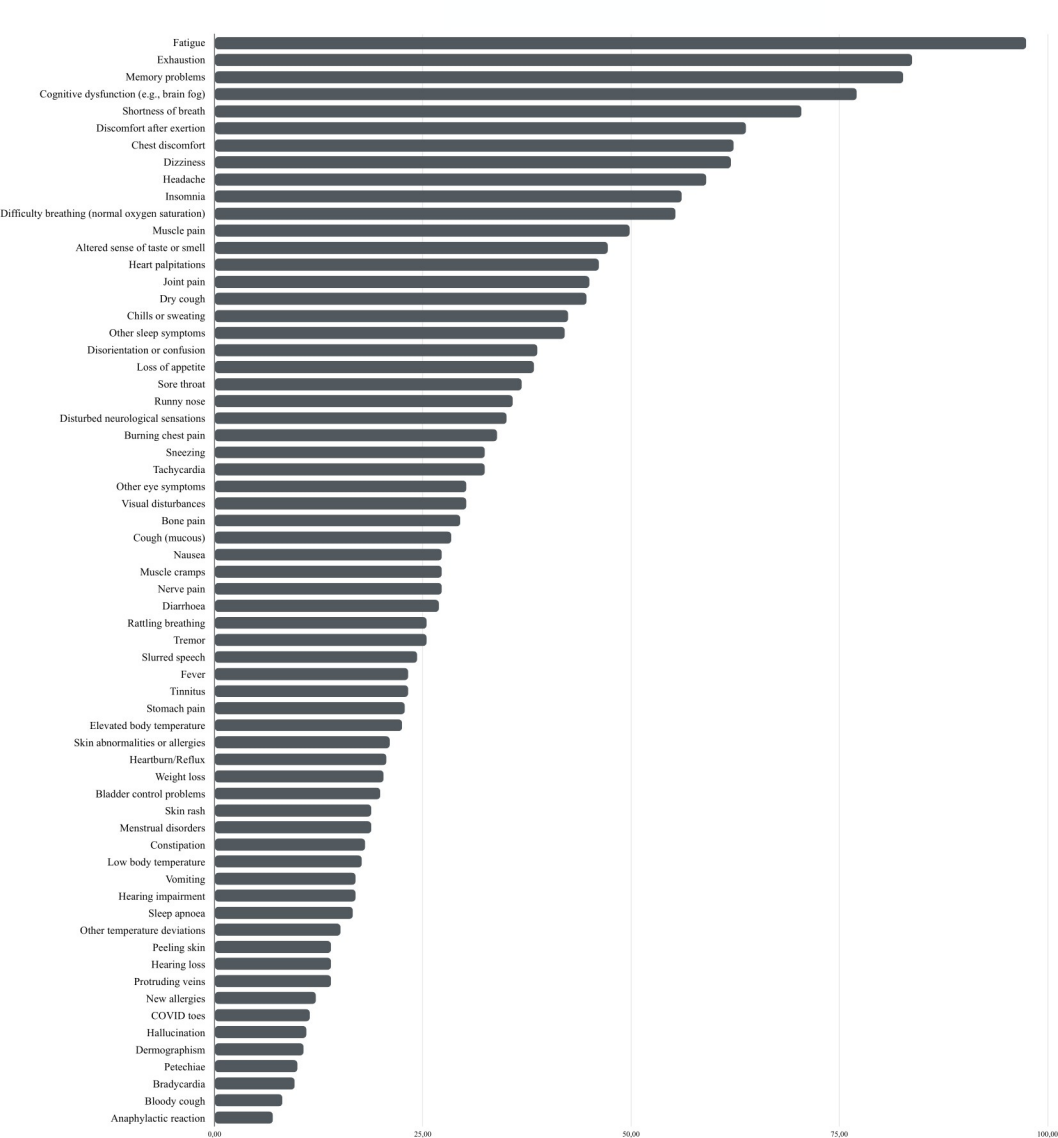
Percent of ongoing or newly developed symptoms (long-COVID symptoms).

A median number of 17.0 (*IQR*: 9–27) symptoms that persisted or were developed after the acute COVID-19 infection were reported. The most prevalent were fatigue (97.4%), exhaustion (83.8%), poor memory (82.7%), cognitive dysfunction (e.g., brain fog; 77.1%), dyspnea (70.5%), discomfort after physical exertion (63.8%), chest discomfort (62.4%), dizziness (62.0%), headache or headache-associated symptoms (59.0%), insomnia (56.1%), breathing difficulties (55.4%), and muscle aches (49.8%). Detailed information about the frequency and burden of all long-COVID symptoms is depicted in S2 Table and Fig 4.

The most significant burden was found to be associated with symptoms such as fatigue (*M* = 4.12, *SD* = 0.99), exhaustion (*M* = 4.03, *SD* = 0.96), discomfort after physical exertion (*M* = 3.92, *SD* = 0.94), sleep alterations (*M* = 3.44, *SD* = 1.16), an altered sense of taste and smell (*M* = 3.44, *SD* = 1.46), and cognitive dysfunction (e.g., brain fog; *M* = 3.43, *SD* = 1.21) (S2 Table). In general, participants experienced a very mild (18.6%), mild (19.4%), moderate (26.1%), strong (20.2%) or very strong (15.7%) burden by their long-COVID symptoms, respectively.

About half of the study participants (49.8%, *n* = 132/265) reported the onset of their long-COVID symptoms within the initial two weeks after the acute infection. 18.9% (*n* = 50) stated that their symptoms emerged between the third- and fourth-week post-infection, while 15.8% (*n* = 42) of the respondents experienced the appearance of ongoing symptoms two- or three months post-infection. A further 15.5% (*n* = 41) reported ongoing symptoms immediately after their infection, after vaccination, or later than three months post-acute infection.

### Extramural examination of long-COVID symptoms

62.3% (*n* = 162/260) underwent medical examinations to clarify their symptoms. These evaluations encompassed pulmonary function tests (72.8%, *n* = 118), X-rays (53.1%, *n* = 86), neurological assessments (41.4%, *n* = 67), MRI scans (38.3%, *n* = 62), CT scans (35.2%, *n* = 57), neuropsychiatric evaluations (25.9%, *n* = 42), and other tests such as ECG (electrocardiogram) or blood tests (18.5%, *n* = 30). A long-COVID outpatient clinic was visited by 17.7% (*n* = 46/260) of all respondents. In addition, 21.9% reported having visited a specialized long-COVID rehabilitation clinic due to their long-COVID symptoms. Numerous study respondents (*n* = 152) further reported connections between their symptoms, for instance, a simultaneous occurrence of anxiety and dyspnea, exhaustion and concentration difficulties, memory problems and headache or between physical exhaustion and cognitive symptoms (e.g., concentration difficulties, or trouble with finding the correct words).

### Alteration of long-COVID symptoms

We further asked all participants about alterations in their long-COVID symptoms by using several multiple-response questions. The majority reported that their symptoms got worse after physical (70.2%, *n* = 186) or mental (52.1%, *n* = 138) activity, while 53.6% (*n* = 142) described them as fluctuating. However, some even reported an improvement in their symptoms after physical (8.3%, *n* = 22) or mental (4.5%, *n* = 12) activity. Moreover, 26.0% (*n* = 69) experienced an overall improvement in their symptoms, while 7.9% (*n* = 21) reported a substantial deterioration of their long-COVID symptoms. A further 28.3% (*n* = 75) described their symptoms as unchanged since their onset. The findings are summarized in Table 5.

**Table 5:** Alterations in long-COVID symptoms (*n* = 265).

Symptoms…	*n*	Percentage (%)
are getting worse after physical activity	186	70.2
are getting worse after mental activity	138	52.1
are fluctuating	142	53.6
are getting better after physical activity	22	8.3
are getting better after mental activity	12	4.5
are improving in general	69	26.0
are getting worse in general	21	7.9
are unchanged since their onset	75	28.3

Percent do not add up to 100% as multiple answers were permitted.

To alleviate the negative impact of long-COVID, participants reported engaging in movement (59.0%, *n* = 138), breathing (53.4%, *n* = 125), and cognitive exercises (36.8%, *n* = 86). Nonetheless, about a quarter (27.4%, *n* = 64) stated having not attempted any exercises yet, although many consider that breathing (65.4%), movement (80.4%), and cognitive exercises (72.1%) could potentially mitigate their symptoms. The participants mentioned a possible enhancement of their overall health as a crucial motivational factor for starting a training program.

### Impact of long-COVID on participant’s professional life

A total of 41.0% (*n* = 133 of 324 respondents) experienced work-related changes since the onset of their long-COVID symptoms. Of those, 9.0% stated to now be unable to work, 23.3% had to reduce working hours, 57.9% went on sick leave, and 25.6% reported other changes in their work routine, such as termination or the requirement for rehabilitation. The median number of work-related sick days was 16 days (*IQR*: 10–42 days). In addition, substantial financial losses were reported by 40.5% of all study participants.

### Impact of long-COVID on participants’ physical and mental health

Regarding breathing difficulties among the survey participants (*n* = 242), 58.7% reported having complete control over their breathing during the day, while the remaining 41.3% stated that control over their breathing is situational. A few (1.1%, *n* = 3) even require oxygen therapy at home. Further, some (23.3%) depend on help from relatives or external organizations in order to perform daily tasks such as cooking, cleaning, childcare responsibilities, or lifting heavy loads.

Additionally, nearly half of all respondents (44.6%, *n* = 116/260) reported a significant increase in substance or medication intake (e.g., alcohol, medication, drugs), and more than a third experienced noticeable changes in their sexual life (e.g., loss of libido; 38.1%, *n* = 88/231). One factor driving these changes was fatigue, as those who experienced substantial changes in their sexual life (*MR* = 130.80, *Mdn* = 5.00) experienced a higher burden of fatigue as compared to those who did not report any sexual alterations (*MR* = 99.29, *Mdn* = 4.00) (*U* = 4188.00, *z* = -3.82, *p* < .001, *r* = -0.26).

Finally, the impact of long-COVID extends beyond physical limitations and encompasses substantial psychological alterations as well. 87.5% (*n* = 231/264) reported a modification in their stress load capacity, as most of these individuals experienced a considerable deterioration (97.0%). Further, mood changes were reported in about 72.1% (*n* = 189/262). Of those, the majority (91.5%) experienced a worsening of their mood since their COVID-19 infection and the associated long-COVID symptoms.

### Vaccination status

134 out of 296 individuals (45.3%) stated to have received a COVID-19 vaccine before their SARS-CoV-2 infection. However, as many study participants got infected prior to the public availability of COVID-19 vaccines in Austria, we further assessed their vaccination status after their infection (when they completed the online survey). 85.2% (*n* = 138/162) of those unvaccinated at the time of their infection got vaccinated after their illness, while 14.8% (*n* = 24) declined to receive a COVID-19 vaccine up to the point of our data collection. The most common vaccine at the first vaccination (*n* = 111) was Comirnaty (BioNTech Pfizer; 60.4%, *n* = 67), followed by Vaxzevria (AstraZeneca; 33.3%, *n* = 37) and Spikevax (Moderna; 6.3%, *n* = 7). A comparable pattern was observable for participants’ second and third vaccination.

### Differences between vaccinated and unvaccinated individuals

Participants who had received at least one dose of the COVID-19 vaccine at the time of their infection (*n* = 128) experienced a significantly shorter acute illness duration (*MR* = 113.72, *Mdn* = 6–10 days) compared to those who were not vaccinated (*n* = 156) prior to their infection (*MR* = 166.11, *Mdn* = 11–15 days; *U* = 6300.50, *z* = -5.52, *p* < .001, *r* = -.33). However, there was no difference between the groups in terms of their self-reported disease course/illness severity (*n* = 289, *χ*^2^(2) = 3.04, *p* = .219, φ_c_ = .10) or hospitalization rates (*n* = 191, *χ*^2^(1) = 0.01, *p* = .904, *OR* = 1.05, 95% CI (0.45, 2.49), φ_c_ = .01).

With respect to work absence, no differences in sick leave days were found between vaccinated (*n* = 115) and unvaccinated (*n* = 127) individuals (*U* = 6364.00, *z* = -1.73, *p* = .084, *r* = -.11). Furthermore, both groups (*n* = 257) did not differ in the frequency of needing support from family, friends, or caregivers (*χ*^2^(1) = 0.50, *p* = .479, *OR* = 1.24, 95% CI (0.69, 2.23), φ_c_ = .04). However, those unvaccinated at the time of their COVID-19 infection more frequently reported having visited a rehabilitation clinic than vaccinated participants (*χ*^2^(1) = 36.11, *p* < .001, *OR* = 14.06, 95% CI (4.90, 40.36), φ_c_ = .37).

Also, the onset of long COVID symptoms (time until new symptoms emerged after the infection) was found to be significantly earlier in vaccinated (*n* = 99, *MR* = 91.85, *Mdn* = after 1–2 weeks) than in unvaccinated (*n* = 122, *MR* = 126.54, *Mdn* = after 3–4 weeks) participants (*U* = 4143.00, *z* = -4.52, *p* < .001, *r* = -.30). Finally, there was no significant difference between the groups regarding the number of their acute (*n* = 295, *U* = 10168.50, *z* = -0.85, *p* = .396, *r* = -.05) or ongoing COVID-19 symptoms (*n* = 265, *U* = 8055.50, *z* = -0.86, *p* = .388, *r* = -.05).

### Differences between male and female respondents

We did not find any statistically significant differences between men and women in the duration of their acute COVID-19 infection (*n* = 284, *U* = 7586.50, *z* = -0.64, *p* = .521, *r* = -.04), self-reported disease course/illness severity (*n* = 289, *χ*^2^(2) = 0.60, *p* = .740, φ_c_ = .05), or hospitalization rates (*n* = 291, *χ*^2^(1) = 3.72, *p* = .054, *OR* = 2.30, 95% CI (0.86, 5.98), φ_c_ = .11). However, the hospitalization analyses indicated a possible trend towards higher hospitalization rates for men (13.0%) compared to women (6.1%).

Further, no differences in the number of sick leave days (*n* = 244, *U* = 4821.00, *z* = -1.72, *p* = .086, *r* = -.11), the frequency of needing support from family, friends, or caregivers (*χ*^2^(1) = 1.20, *p* = .273, *OR* = 1.47, 95% CI (0.74, 2.91), φ_c_ = .07), the frequency of having visited a rehabilitation clinic (*χ*^2^(1) = 0.09, *p* = .767, *OR* = 0.91, 95% CI (0.47, 1.75), φ_c_ = .02), or the onset of long-COVID symptoms (*n* = 220, *U* = 5336.00, *z* = 1.74, *p* = .081, *r* = .12) were observed.

Concerning the frequency of symptoms during the acute phase of the illness, no significant difference was observed between men and women as well (*U* = 8247.50, *z* = -0.34, *p* = .731, *r* = —.02). However, in general, women (*MR* = 141.39, *Mdn* = 18 symptoms) reported significantly more ongoing COVID-19 (long-COVID) symptoms compared to men (*MR* = 110.98, *Mdn* = 13 symptoms) (*U* = 5242.50, *z* = -2.83, *p* = .005, *r* = -.17).

### Possible risk factors associated with acute COVID-19 and long-COVID symptoms

Next, we were interested in possible risk factors (BMI, age, and hypertension) associated with participants’ acute infection and ongoing symptoms. A higher BMI (*M* = 26.19, *SD* = 6.73) was associated with a longer duration of illness during the initial COVID-19 infection (*r*_*s*_ = .13, *p* = .025).

Further analyses confirmed that individuals with different self-reported disease courses (asymptomatic (*n* = 17), mild (*n* = 210), severe (*n* = 56)), on average, differed in their body mass index (*F*(2, 280) = 5.50, *p* = .005, η_p_^2^ = .038). We found significant differences between individuals with a mild (*M* = 25.46 kg/m^2^, *SD* = 6.55) and severe (*M* = 28.71 kg/m^2^, *SD* = 7.29) illness (*M*_diff_ = 3.25 kg/m^2^, *p* = .004), while no differences to asymptomatic individuals (*M* = 27.15 kg/m^2^, *SD* = 5.37) were observable (asymptomatic vs. mild: *p* = .675; asymptomatic vs. severe: *p* = .778).

In addition, no age differences between individuals with different disease courses (asymptomatic (*n* = 17), mild (*n* = 202), or severe (*n* = 57)) were found in this data (*F*(2, 273) = 1.90, *p* = .151, η_p_^2^ = .014), but our analyses revealed a positive relationship between age (*M* = 44.69, *SD* = 12.81) and participants’ illness duration during their acute infection (*r*_*s*_ = .23, *p* < .001). However, age was not associated with the frequency of acute COVID-19 symptoms (*r* = .02, *p* = .713) and long-COVID symptoms (*r* = .03, *p* = .627).

Among 340 participants that made a statement about their blood pressure, 8.8% (*n* = 30) suffered from hypertension. Participants with heightened blood pressure (*MR* = 159.25, *Mdn* = 11–15 days) did not statistically differ in the duration of their acute illness from normotensive participants at the time of completing the survey (*MR* = 143.47, *Mdn* = 11–15 days) (*n* = 289, *U* = 4053.00, *z* = 0.98, *p* = .327, *r* = .06). In addition, no statistically significant differences between those with and without hypertension were found regarding their self-reported illness severity (asymptomatic, mild, severe; *n* = 294, *χ*^2^(2) = 5.59, *p* = .061, φ_c_ = .14), the frequency of symptoms during their acute illness (*U* = 3959.00, *z* = -1.35, *p* = .178, *r* = -.07) or ongoing symptoms (*U* = 4329.50, *z* = -0.63, *p* = .531, *r* = -.03), and the time until the occurrence of long-COVID symptoms (*U* = 2274.00, *z* = -1.09, *p* = .275, *r* = -.07).

## Discussion

The findings of the present online study offer valuable insight into the various domains impacted by individuals suffering from long-COVID. Symptoms such as fatigue, exhaustion, and considerable cognitive deficits (e.g., brain fog or memory problems) were predominant in both the acute and ongoing phases of their illness. This clinical presentation is consistent with other studies, indicating that long-COVID symptoms affect multiple organ systems, with cognitive dysfunction and fatigue being among the most frequent ongoing symptoms [20]. Although those symptoms were already reported in other studies [11, 20], our findings suggest that they were experienced as a significant burden for those affected, which might lead to serious mood disorders such as depression or anxiety [29]. Thus, highlighting the burden and impact of long-COVID and focusing on tailored rehabilitation opportunities represents a significant challenge for future research on this topic. We could further confirm a shorter acute illness duration and an earlier onset of long-COVID symptoms in vaccinated compared to unvaccinated individuals, as well as a higher number of long-COVID symptoms in women than in men. The key findings of the present study are summarized in the following section and compared to the current state of knowledge.

### Acute COVID-19 illness

Consistent with one of the earliest studies investigating the course of a coronavirus infection [2], reporting about 81% of mild and 19% of severe/critical cases, most participants in the present study stated to have experienced a mild (74.3%), severe (19.7%) or asymptomatic (6.0%) infection. In line with Menni et al. [30], the acute illness of most individuals did not exceed about two weeks. Symptoms affecting the respiratory tract (e.g., cough, dyspnea, chest tightness) as well as fatigue/exhaustion, fever, headache, or cognitive dysfunction were present as typical signs of an acute COVID-19 infection [11, 31, 32]. Our results further suggest that both a higher body mass index and a higher age are associated with a longer-lasting illness during the acute infection. A higher BMI, on average, was further present in those with a more significant illness severity [33, 34]. In addition, diverging illness characteristics of vaccinated (45.3%) and unvaccinated (54.7%) individuals (at the time of their infection) were present, as not being vaccinated was associated with a longer-lasting acute infection. Finally, although belonging to the male sex was found to be associated with an increased risk of hospitalization in previous studies [35], no significant gender differences in hospitalization rates were present in the current study. Nonetheless, we found indications that men were hospitalized about twice as often as women (13.0% vs. 6.1%).

### Long-COVID

In the majority (49.8%), ongoing or newly developed symptoms emerged already in the first two weeks post-infection. Common symptoms included fatigue, exhaustion, memory problems, cognitive dysfunction, and dyspnea. Consequently, numerous participants reported an adverse impact regarding their profession (41.0%), financial losses (40.5%), and changes in their ability to cope with stress (87.5%). Those affected encountered changes in their work life such as sick leave, loss of income, and reduced working hours or were even incapable of work which also aligns with prior literature [11, 20]. Ziauddeen et al., for instance, reported a loss of income in 37.6% of all study participants [11], comparable to a high number of 40.5% of participants in the current study.

Furthermore, long-COVID symptoms such as cognitive dysfunction or brain fog [20, 36, 37] might interfere with demands in the work and private life of those affected, as those symptoms were often described as fluctuating and deteriorating after physical or cognitive activity [11]. Consequently, reducing working hours has been a common outcome for those suffering from long-COVID [11, 20]. A gradual reintegration into the labor market as well as adjusted working hours might be advisable to prevent prolonged sick leave or early retirement.

It is concerning to note that a considerable number of participants in the current study experienced changes in their mood (72.1%), had problems with their breathing control (41.3%), were dependent on help from organizations or relatives (23.3%) or suffered from changes in their sexual life (38.1%). Alterations in the sexual life (e.g., erectile dysfunction) of patients living with long-COVID had also been reported in previous studies [24], which might lead to significant distress. Psychological distress and depressive symptoms were found to be prevalent in more than 25% of individuals three months after the acute phase of infection [38], suggesting that the risk of mood disorders such as depression or anxiety in COVID-19 survivors is high [39]. Comprehensive mental health care and clinical strategies for individuals with long-COVID are therefore needed.

Regarding the influence of COVID vaccines on the emergence of long-COVID or its symptoms, studies consistently show an association between vaccinations and reduced odds and risk of developing long-COVID [40, 41]. Consequently, ongoing research proposes that COVID-19 vaccines could offer both protective and therapeutic benefits against long-COVID [42]. Although numerous studies provided evidence that the risk of developing long-COVID is lower among vaccinated individuals than in those without a vaccination [14, 15, 43], there seems to be no difference regarding the number of long-COVID symptoms between both groups according to the results found in this study. However, with respect to gender differences, we did observe a higher number of long-COVID symptoms in women compare to men, which aligns with the findings of a previous study by Jensen et al. [44].

Despite the range of symptoms and adverse impact on various domains, most individuals did not seek help in long-COVID outpatient clinics (82.3%) or rehabilitation facilities (78.1%), which might result in a significant economic burden on the worldwide healthcare system and the well-being of those affected.

### Limitations

The current study used a convenience non-probability sampling approach, whereby subjects were chosen not randomly from the population but rather based on their geographical proximity and availability at a given time. In addition, the majority were female and reported a high educational status. Nonetheless, studies have demonstrated that long-COVID occurs more frequently in women, which makes it reasonable for a higher representation of women in long-COVID studies [45]. Given that this study was conducted online, it might not have been accessible to all individuals living with long-COVID in the population. Consequently, the generalization of our results to the total population is limited.

Due to economic reasons, the length of the survey was as short as possible in order to recruit a large number of participants with a little drop-out rate of unanswered questions. Therefore, some parameters (e.g., the Post-COVID-19 functional status [46]) were not collected and should be included in future studies as they might add valuable information to this research topic. In addition, the current study may be affected by two possible sources of bias: Firstly, participants may have had difficulty correctly recalling their symptoms, burden, or the length of their acute illness, especially if their initial infection occurred several months before completing the online survey. In addition, individuals with more severe symptoms might have been more likely to participate in a long-COVID online survey, again compromising the generalizability of the results.

Also, despite enquiring the duration of the acute disease, and the timepoint of the first long-COVID symptoms, we cannot guarantee that all participants meet the current definition of long-COVID with persistence of symptoms being present for at least four weeks following the acute infection. Finally, this study did not provide guidelines for categorizing individuals into “asymptomatic”, “mild”, and”severe” disease courses. Therefore, participants could indicate a severe disease course, for instance, regardless of whether they were hospitalized or not.

### Implications for future research

Future studies should raise awareness concerning the effects of COVID-19 tailored rehabilitation possibilities. However, as general treatment for patients is scarce or unavailable [7], multidisciplinary teams need to specialize in rehabilitating the various symptoms associated with this condition. As fatigue (ongoing and constant exhaustion in mental and physical aspects that does not improve with resting/sleeping) and exhaustion (short-term lack of energy that improves after taking a rest), for instance, emerged among the most prevalent symptoms reported, future research should focus on validating the use of techniques such as psychoeducation [47], energy management [47], training based on cognitive-behavioral-therapy [48, 49], mindfulness [50] or relaxation exercises [51] in ameliorating fatigue in patients suffering from long-COVID. In addition, cognitive deficits like memory problems, trouble finding the correct words, and planning-oriented thinking [52, 53] are common long-COVID symptoms and require tailored cognitive rehabilitation programs. Future research on long-COVID should also examine the implications and consequences due to insufficient, incomplete, or discontinued treatment in the acute phase, since a variety of consequences might be associated with it.

## Conclusion

Long-COVID represents a highly heterogeneous disease, encompassing a variety of symptoms such as fatigue, cognitive dysfunction, or dyspnea. The impact of this prolonged illness is not yet fully understood, as symptoms can affect multiple domains, including an individuals’ professional and personal life. Multidisciplinary teams and treatments are needed to develop individually tailored rehabilitation approaches, enabling individuals to ameliorate their symptoms and better cope with the substantial burden of long-COVID in the future.

## Supporting information

S1 Table Frequency of acute symptoms and subjective burden (n = 302). *a* Multiple modes exist, the smallest value is shown.
(PDF)

S2 Table Frequency of ongoing symptoms and subjective burden (n = 271). *a* Multiple modes exist, the smallest value is shown.
(PDF)

S1 File Research data. Koini, Marisa. Characteristics and burden of acute COVID-19 and long-COVID. Ann Arbor, MI: Inter-university Consortium for Political and Social Research [distributor], 2024-01-06. https://doi.org/10.3886/E196861V1.
(XLSX)

## References

[1] World Health Organization: WHO COVID-19 Dashboard. 2022 [cited April 4, 2023]. Available from: https://covid19.who.int

[2] Wu Z, McGoogan JM. Characteristics of and Important Lessons From the Coronavirus Disease 2019 (COVID-19) Outbreak in China. JAMA. 2020;323(13):1236–1242. doi: 10.1001/jama.2020.2648 32091533

[3] Katzenschlager S, Zimmer AJ, Gottschalk C, Grafeneder J, Schmitz S, Kraker S, et al. Can we predict the severe course of COVID-19—a systematic review and meta-analysis of indicators of clinical outcome?. PLOS ONE. 2021;16(7):e0255154. doi: 10.1371/journal.pone.0255154 34324560 PMC8321230

[4] Borges do Nascimento IJ, Cacic N, Abdulazeem HM, von Groote TC, Jayarajah U, Weerasekara I, et al. Novel Coronavirus Infection (COVID-19) in Humans: A Scoping Review and Meta-Analysis. Journal of Clinical Medicine. 2020;9(4):941. doi: 10.3390/jcm9040941 32235486 PMC7230636

[5] Alimohamadi Y, Sepandi M, Taghdir M, Hosamirudsari H. Determine the most common clinical symptoms in COVID-19 patients: a systematic review and meta-analysis. Journal of Preventive Medicine and Hygiene. 2020;61(3):E304–E312. doi: 10.15167/2421-4248/jpmh2020.61.3.1530 33150219 PMC7595075

[6] Haslam A, Thibault O, Prasad V. The definition of long COVID used in interventional studies. European Journal of Clinical Investigation. 2023;53(8). doi: 10.1111/eci.13989 36964995

[7] Koczulla AR, Ankermann T, Behrends U, Berlit P, Böing S, Brinkmann F, et al. S1-Leitlinie Post-COVID/Long-COVID. Pneumologie. 2021;75(11):869–900. doi: 10.1055/a-1551-9734 34474488

[8] Ora J, Calzetta L, Frugoni C, Puxeddu E, Rogliani P. Expert Guidance on the Management and Challenges of Long-COVID Syndrome: A Systematic review. Expert Opinion on Pharmacotherapy. 2023;24(3):315–30. doi: 10.1080/14656566.2022.2161365 36542805

[9] COVID-19 rapid guideline: managing the long-term effects of COVID-19. 2022 National Institute for Health and Care Excellence (NICE) [cited November 16, 2023]. Available from: https://www.nice.org.uk/guidance/ng188/resources 33555768

[10] Daroische R, Hemminghyth MS, Eilertsen TH, Breitve MH, Chwiszczuk LJ. Cognitive Impairment After COVID-19—A Review on Objective Test Data. Frontiers in Neurology. 2021;(12):1–9. doi: 10.3389/fneur.2021.699582 34393978 PMC8357992

[11] Ziauddeen N, Gurdasani D, O’Hara ME, Hastie C, Roderick P, Yao G, et al. Characteristics and impact of Long Covid: Findings from an online survey. Sutcliffe CG, editor. PLOS ONE. 2022;17(3):e0264331. doi: 10.1371/journal.pone.0264331 35259179 PMC8903286

[12] Reuken PA, Scherag A, Stallmach A. Postcoronavirus Disease Chronic Fatigue Is Frequent and Not Only Restricted to Hospitalized Patients. Critical Care Medicine. 2021;49(10):e1052–3. doi: 10.1097/CCM.0000000000005122 34034301 PMC8439636

[13] Subramanian A, Nirantharakumar K, Hughes S, Myles P, Williams T, Gokhale KM, et al. Symptoms and risk factors for long COVID in non-hospitalized adults. Nature Medicine. 2022;28(8):1706–14. doi: 10.1038/s41591-022-01909-w 35879616 PMC9388369

[14] Thompson EJ, Williams DM, Walker AJ, Mitchell RE, Niedzwiedz CL, Yang TC, et al. Long COVID burden and risk factors in 10 UK longitudinal studies and electronic health records. Nature Communications. 2022;13(1):3528. doi: 10.1038/s41467-022-30836-0 35764621 PMC9240035

[15] Notarte KI, Catahay JA, Velasco JV, Pastrana A, Ver AT, Pangilinan FC, et al. Impact of COVID-19 vaccination on the risk of developing long-COVID and on existing long-COVID symptoms: A systematic review. eClinicalMedicine. 2022;(53):1–19. doi: 10.1016/j.eclinm.2022.101624 36051247 PMC9417563

[16] Vimercati L, De Maria L, Quarato M, Caputi A, Gesualdo L, Migliore G, et al. Association between Long COVID and Overweight/Obesity. Journal of Clinical Medicine. 2021;10(18):4143. doi: 10.3390/jcm10184143 34575251 PMC8469321

[17] Cazé AB, Cerqueira‐Silva T, Bomfim AP, De Souza GL, Azevedo ACAC, Brasil MQ, et al. Prevalence and risk factors for long COVID after mild disease: A cohort study with a symptomatic control group. Journal of Global Health. 2023;13. doi: 10.7189/jogh.13.06015 37166260 PMC10173895

[18] Cabrera Martimbianco AL, Pacheco RL, Bagattini ÂM, Riera R. Frequency, signs and symptoms, and criteria adopted for long COVID‐19: A systematic review. International Journal of Clinical Practice. 2021;75(10):e14357. doi: 10.1111/ijcp.14357 33977626 PMC8236920

[19] Venkatesh V. Impacts of COVID-19: A research agenda to support people in their fight. International Journal of Information Management. 2020;(55):102197. doi: 10.1016/j.ijinfomgt.2020.102197 32836648 PMC7368151

[20] Davis HE, Assaf GS, McCorkell L, Wei H, Low RJ, Re’em Y, et al. Characterizing long COVID in an international cohort: 7 months of symptoms and their impact. EClinicalMedicine. 2021;(38):101019. doi: 10.1016/j.eclinm.2021.101019 34308300 PMC8280690

[21] Lakhan R, Agrawal A, Sharma M. Prevalence of depression, anxiety, and stress during COVID-19 pandemic. Journal of Neurosciences in Rural Practice. 2020;11(4):519–525. doi: 10.1055/s-0040-1716442 33144785 PMC7595780

[22] Lamontagne SJ, Winters MF, Pizzagalli DA, Olmstead MC. Post-acute sequelae of COVID-19: Evidence of mood & cognitive impairment. Brain, Behavior, & Immunity–Health. 2021;(17):100347. doi: 10.1016/j.bbih.2021.100347 34549199 PMC8437695

[23] Rudenstine S, Schulder T, Bhatt KJ, McNeal K, Ettman CK, Galea S. Long-COVID and comorbid depression and anxiety two years into the COVID-19 pandemic. Psychiatry Research. 2022;(317):114924. doi: 10.1016/j.psychres.2022.114924 37732865 PMC9597528

[24] Harirugsakul K, Wainipitapong S, Phannajit J, Paitoonpong L, Tantiwongse K. Erectile dysfunction after COVID-19 recovery: A follow-up study. Kim T, editor. PLOS ONE. 2022;17(10):e0276429. doi: 10.1371/journal.pone.0276429 36264947 PMC9584530

[25] Asadi‐Pooya AA, Akbari A, Emami A, Lotfi M, Rostamihosseinkhani M, Nemati H, et al. Long COVID syndrome‐associated brain fog. Journal of Medical Virology. 2021;94(3). doi: 10.1002/jmv.27404 34672377 PMC8662118

[26] Lopez-Leon S, Wegman-Ostrosky T, Perelman C, Sepulveda R, Rebolledo PA, Cuapio A, et al. More than 50 long-term effects of COVID-19: a systematic review and meta-analysis. Scientific Reports. 2021;11(1):16144. doi: 10.1038/s41598-021-95565-8 34373540 PMC8352980

[27] Zawilska JB, Kuczyńska K. Psychiatric and neurological complications of long COVID. Journal of Psychiatric Research. 2022;(156):349–360. doi: 10.1016/j.jpsychires.2022.10.045 36326545 PMC9582925

[28] Sansone A, Mollaioli D, Limoncin E, Ciocca G, Bắc NH, Cao TN, et al. The Sexual Long COVID (SLC): erectile dysfunction as a biomarker of systemic complications for COVID-19 long haulers. Sexual Medicine Reviews. 2021;10(2):271–285. doi: 10.1016/j.sxmr.2021.11.001 34933829 PMC8604714

[29] Mazza MG, Palladini M, Poletti S, Benedetti F. Post-COVID-19 Depressive Symptoms: Epidemiology, Pathophysiology, and Pharmacological Treatment. CNS Drugs. 2022;36(7):681–702. doi: 10.1007/s40263-022-00931-3 35727534 PMC9210800

[30] Menni C, Valdes AM, Polidori L, Antonelli M, Penamakuri S, Nogal A, et al. Symptom prevalence, duration, and risk of hospital admission in individuals infected with SARS-CoV-2 during periods of omicron and delta variant dominance: a prospective observational study from the ZOE COVID Study. The Lancet. 2022;399(10335):1618–1624. doi: 10.1016/S0140-6736(22)00327-0 35397851 PMC8989396

[31] Pascarella G, Strumia A, Piliego C, Bruno F, Del Buono R, Costa F, et al. COVID‐19 diagnosis and management: a comprehensive review. Journal of Internal Medicine. 2020;288(2):157–265. doi: 10.1111/joim.13091 32348588 PMC7267177

[32] Unim B, Palmieri L, Lo Noce C, Brusaferro S, Onder G. Prevalence of COVID-19-related symptoms by age group. Aging Clinical and Experimental Research. 2021;33(4):1145–7. doi: 10.1007/s40520-021-01809-y 33650071 PMC7919615

[33] Peres KC, Riera R, Martimbianco ALC, Ward LS, Cunha LL. Body Mass Index and Prognosis of COVID-19 Infection. A Systematic Review. Frontiers in Endocrinology. 2020;(11):1–10. doi: 10.3389/fendo.2020.00562 32922366 PMC7456965

[34] Kwok S, Adam S, Ho JH, Iqbal Z, Turkington P, Razvi S, et al. Obesity: A critical risk factor in the COVID ‐19 pandemic. Clinical Obesity. 2020;10(6):e12403. doi: 10.1111/cob.12403 32857454 PMC7460880

[35] Gomez JMD, Du-Fay-de-Lavallaz JM, Fugar S, Sarau A, Simmons JA, Clark B, et al. Sex Differences in Coronavirus Disease 2019 (COVID-19) Hospitalization and Mortality. Journal of Women’s Health. 2021;30(5):646–653. doi: 10.1089/jwh.2020.8948 33826864

[36] Graham EL, Clark JR, Orban ZS, Lim PH, Szymanski AL, Taylor C, et al. Persistent neurologic symptoms and cognitive dysfunction in non‐hospitalized Covid‐19 “long haulers.” Annals of Clinical and Translational Neurology. 2021;8(5):1073–1085. doi: 10.1002/acn3.51350 33755344 PMC8108421

[37] Delgado-Alonso C, Valles-Salgado M, Delgado-Álvarez A, Yus M, Gómez-Ruiz N, Jorquera M, et al. Cognitive dysfunction associated with COVID-19: A comprehensive neuropsychological study. Journal of Psychiatric Research. 2022;(150):40–46. doi: 10.1016/j.jpsychires.2022.03.033 35349797 PMC8943429

[38] Bautista-Rodriguez E, Cortés-Álvarez NY, Vuelvas-Olmos CR, Reyes-Meza V, González-López T, Flores-delosÁngeles C, et al. Stress, anxiety, depression and long COVID symptoms. Fatigue: Biomedicine, Health & Behavior. 2022;11(1):35–54. doi: 10.1080/21641846.2022.2154500

[39] Fahriani M, Ilmawan M, Fajar JK, Maliga HA, Frediansyah A, Masyeni S, et al. Persistence of long COVID symptoms in COVID-19 survivors worldwide and its potential pathogenesis—A systematic review and meta-analysis. Narra J. 2021;1(2):e36. doi: 10.52225/narraj.v1i2.36 PMC10914031 38449463

[40] Brannock MD, Chew R, Preiss A, Hadley E, Redfield S, McMurry JA, et al. Long COVID risk and pre-COVID vaccination in an EHR-based cohort study from the RECOVER program. Nature Communications. 2023;14(1). doi: 10.1038/s41467-023-38388-7 37217471 PMC10201472

[41] Gao P, Liu J, M L. Effect of COVID-19 vaccines on reducing the risk of long COVID in the real world: A Systematic Review and Meta-Analysis. International Journal of Environmental Research and Public Health. 2022;19(19):12422. doi: 10.3390/ijerph191912422 36231717 PMC9566528

[42] Byambasuren O, Stehlik P, Clark J, Alcorn K, Glasziou P. Effect of covid-19 vaccination on long covid: systematic review. BMJ Medicine. 2023;2(1):e000385. doi: 10.1136/bmjmed-2022-000385 36936268 PMC9978692

[43] Ayoubkhani D, Bermingham C, Pouwels KB, Glickman M, Nafilyan V, Zaccardi F, et al. Trajectory of long covid symptoms after covid-19 vaccination: community based cohort study. BMJ. 2022;(377):e069676. doi: 10.1136/bmj-2021-069676 35584816 PMC9115603

[44] Jensen A, Castro AW, Feretti MT, Martinkova J, Vasilevskaya A, Chadha AS, et al. Sex and gender differences in the neurological and neuropsychiatric symptoms of long COVID: a narrative review. Italian Journal of Gender-Specific Medicine. 2022;8(1):18–28. doi: 10.1723/3769.37563

[45] Krishna BA, Wills MR, Sithole N. Long COVID: what is known and what gaps need to be addressed. British Medical Bulletin. 2023;147(1):6–19. doi: 10.1093/bmb/ldad016 37434326 PMC10502447

[46] Klok FA, Boon GJAM, Barco S, Endres M, Geelhoed JJM, Knauss S, et al. The Post-COVID-19 Functional Status scale: a tool to measure functional status over time after COVID-19. The European Respiratory Journal. 2020;56(1):2001494. doi: 10.1183/13993003.01494-2020 32398306 PMC7236834

[47] Flachenecker P, Meißner H. Fatigue bei Multipler Sklerose–wie diagnostizieren, wie behandeln?. Neurologie & Rehabilitation. 2014;20(5):273–281.

[48] Neill J, Belan I, Ried K. Effectiveness of non-pharmacological interventions for fatigue in adults with multiple sclerosis, rheumatoid arthritis, or systemic lupus erythematosus: a systematic review. Journal of Advanced Nursing. 2006;56(6):617–35. doi: 10.1111/j.1365-2648.2006.04054.x 17118041

[49] Harrison AM, Safari R, Mercer T, Picariello F, van der Linden ML, White C, et al. Which exercise and behavioural interventions show most promise for treating fatigue in multiple sclerosis? A network meta-analysis. Multiple Sclerosis Journal. 2021;27(11):1657–1678. doi: 10.1177/1352458521996002 33876986 PMC8474304

[50] Grossman P, Kappos L, Gensicke H, D’Souza M, Mohr DC, Penner IK, et al. MS quality of life, depression, and fatigue improve after mindfulness training: A randomized trial. Neurology. 2010;75(13):1141–1149. doi: 10.1212/WNL.0b013e3181f4d80d 20876468 PMC3463050

[51] Dayapoğlu N, Tan M. Evaluation of the Effect of Progressive Relaxation Exercises on Fatigue and Sleep Quality in Patients with Multiple Sclerosis. The Journal of Alternative and Complementary Medicine. 2012;18(10):983–987. doi: 10.1089/acm.2011.0390 22967281 PMC3469207

[52] Alemanno F, Houdayer E, Parma A, Spina A, Del Forno A, Scatolini A, et al. COVID-19 cognitive deficits after respiratory assistance in the subacute phase: A COVID-rehabilitation unit experience. Di Gennaro F, editor. PLOS ONE. 2021;16(2):e0246590. doi: 10.1371/journal.pone.0246590 33556127 PMC7870071

[53] Pistarini C, Fiabane E, Houdayer E, Vassallo C, Manera MR, Alemanno F. Cognitive and Emotional Disturbances Due to COVID-19: An Exploratory Study in the Rehabilitation Setting. Frontiers in Neurology. 2021;(12):1–8. doi: 10.3389/fneur.2021.643646 34079511 PMC8165252

