# Supplementary material for: Characteristics and burden of acute COVID-19 and long-COVID: Demographic, physical, mental health, and economic perspectives
Source: PLoS One. 2024 Jan 22;19(1):e0297207. doi: 10.1371/journal.pone.0297207 (PMC10802963; doi:10.1371/journal.pone.0297207)
Supplement: S1 Table — a Multiple modes exist, the smallest value is shown. (PDF) [file pone.0297207.s001.pdf]

Supplement Table 1

Frequency of acute symptoms and subjective burden (n = 302)

Symptom	Subjective burden					Sum	%	Subjective burden [1-5]			
	Very mild	Mild	Moderate	Strong	Very strong			*M*	*SD*	*Mdn*	*Mode*
Fatigue	7	22	45	109	99	282	93.38	3.96	1.02	4	4
Exhaustion	3	14	38	93	107	255	84.44	4.13	0.94	4	5
Dry cough	32	21	52	71	54	230	76.16	3.41	1.32	4	4
Cognitive dysfunction (e.g., brain fog)	36	44	59	58	31	228	75.50	3.02	1.28	3	3
Memory problems	41	45	81	40	20	227	75.17	2.79	1.19	3	3
Runny nose	57	47	48	43	28	223	73.84	2.72	1.36	3	1
Loss of appetite	36	38	61	49	35	219	72.52	3.04	1.30	3	3
Headache	10	27	42	66	74	219	72.52	3.76	1.18	4	5
Fever	63	46	51	35	23	218	72.19	2.58	1.34	2.5	1
Chills or sweating	20	24	72	56	45	217	71.85	3.38	1.20	3	3
Muscle pain	14	29	58	59	57	217	71.85	3.53	1.20	4	4
Shortness of breath	15	25	56	56	63	215	71.19	3.59	1.22	4	5
Sore throat	39	41	62	35	29	206	68.21	2.87	1.30	3	3
Chest discomfort	25	26	56	53	46	206	68.21	3.33	1.29	3	3

Difficulty breathing	36	26	47	49	43	201	66.56	3.18	1.39	3	4
Elevated body temperature	55	33	49	33	28	198	65.56	2.73	1.40	3	1
Altered sense of taste or smell	17	15	24	42	99	197	65.23	3.97	1.31	5	5
Dizziness	19	35	46	55	37	192	63.58	3.29	1.25	3	4
Discomfort after exertion	5	11	52	55	56	179	59.27	3.82	1.04	4	5
Sneezing	41	34	51	31	16	173	57.28	2.69	1.27	3	3
Joint pain	14	20	40	47	48	169	55.96	3.56	1.25	4	5
Rattling breathing	59	30	33	25	12	159	52.65	2.38	1.33	2	1
Insomnia	25	17	41	37	32	152	50.33	3.22	1.35	3	3
Other sleep symptoms	25	21	33	42	30	151	50.00	3.21	1.36	3	4
Cough (mucous)	37	25	37	28	20	147	48.68	2.79	1.37	3	1[a]
Heart palpitations	19	21	39	41	27	147	48.68	3.24	1.28	3	4
Diarrhoea	52	32	29	18	9	140	46.36	2.29	1.27	2	1
Nausea	43	23	31	22	10	129	42.72	2.48	1.32	2	1
Bone pain	23	20	27	35	23	128	42.38	3.12	1.37	3	4
Burning chest pain	30	23	30	27	14	124	41.06	2.77	1.34	3	1[a]
Disorientation or confusion	45	27	24	20	6	122	40.40	2.30	1.26	2	1
Tachycardia	32	25	29	25	9	120	39.74	2.62	1.29	3	1
Weight loss	38	26	24	18	7	113	37.42	2.38	1.27	2	1
Other eye symptoms	33	21	34	13	11	112	37.09	2.54	1.29	3	3

Stomach pain	57	17	16	12	4	106	35.10	1.95	1.22	1	1
Vomiting	69	17	12	4	3	105	34.77	1.62	1.02	1	1
Nerve pain	27	19	23	22	14	105	34.77	2.78	1.39	3	1
Disturbed neurological sensations	21	22	35	15	11	104	34.44	2.74	1.24	3	3
Muscle cramps	36	23	17	12	9	97	32.12	2.33	1.34	2	1
Tinnitus	28	19	19	20	9	95	31.46	2.61	1.36	3	1
Tremor	33	21	24	10	6	94	31.13	2.31	1.24	2	1
Visual disturbances	28	24	27	10	5	94	31.13	2.36	1.17	2	1
Low body temperature	51	14	12	6	4	87	28.81	1.83	1.18	1	1
Heartburn/Reflux	39	23	11	10	4	87	28.81	2.05	1.21	2	1
Sleep apnea	39	10	16	12	4	81	26.82	2.16	1.31	2	1
Skin rash	38	14	12	12	5	81	26.82	2.16	1.33	2	1
Slurred speech	33	19	18	7	2	79	26.16	2.06	1.11	2	1
Hearing impairment	34	19	16	2	3	74	24.50	1.93	1.08	2	1
Constipation	43	12	12	3	3	73	24.17	1.78	1.12	1	1
Bladder control problems	45	11	9	6	2	73	24.17	1.75	1.13	1	1
Skin abnormalities or allergies	36	8	11	10	6	71	23.51	2.18	1.41	1	1
Peeling skin	41	10	10	5	3	69	22.85	1.83	1.19	1	1
Hearing loss	40	12	11	4	2	69	22.85	1.78	1.10	1	1
COVID toes	46	5	7	8	2	68	22.52	1.75	1.21	1	1

Menstrual disorders	33	6	15	6	7	67	22.19	2.22	1.41	2	1
Other temperature deviations	37	10	11	4	3	65	21.52	1.86	1.18	1	1
Bradycardia	47	9	5	0	4	65	21.52	1.54	1.08	1	1
Protruding veins	38	10	9	6	1	64	21.19	1.78	1.11	1	1
Bloody cough	52	2	2	5	0	61	20.20	1.34	0.89	1	1
Hallucinations	51	4	3	2	1	61	20.20	1.33	0.85	1	1
Petechial rash	39	7	9	2	3	60	19.87	1.72	1.15	1	1
New allergies	43	4	5	1	2	55	18.21	1.45	1.00	1	1
Dermographism	37	6	6	2	3	54	17.88	1.67	1.17	1	1
Anaphylactic reaction	45	2	3	2	0	52	17.22	1.27	0.74	1	1

[*] *Note.* [a] Multiple modes exist, the smallest value is shown
