# Supplementary material for: Characteristics and burden of acute COVID-19 and long-COVID: Demographic, physical, mental health, and economic perspectives
Source: PLoS One. 2024 Jan 22;19(1):e0297207. doi: 10.1371/journal.pone.0297207 (PMC10802963; doi:10.1371/journal.pone.0297207)
Supplement: S2 Table — a Multiple modes exist, the smallest value is shown. (PDF) [file pone.0297207.s002.pdf]

Supplement Table 2

Frequency of ongoing symptoms and subjective burden (n = 271)

Symptom	Subjective burden					Sum	%	Subjective burden [1-5]			
	Very mild	Mild	Moderate	Strong	Very strong			*M*	*SD*	*Mdn*	*Mode*
Fatigue	8	10	37	97	112	264	97.42	4.12	0.99	4	5
Exhaustion	3	11	49	77	87	227	83.76	4.03	0.96	4	5
Memory problems	15	34	76	66	33	224	82.66	3.30	1.10	3	3
Cognitive dysfunction (e.g., brain fog)	16	31	58	56	48	209	77.12	3.43	1.21	3	3
Shortness of breath	14	37	50	58	32	191	70.48	3.30	1.17	3	4
Discomfort after exertion	0	13	44	59	57	173	63.84	3.92	0.94	4	4
Chest discomfort	23	28	47	41	30	169	62.36	3.16	1.28	3	3
Dizziness	20	43	42	33	30	168	61.99	3.06	1.28	3	2
Headache	9	28	49	41	33	160	59.04	3.38	1.16	3	3
Insomnia	16	26	41	40	29	152	56.09	3.26	1.25	3	3
Difficulty breathing	12	22	53	43	20	150	55.35	3.25	1.11	3	3
Muscle pain	11	19	41	37	27	135	49.82	3.37	1.19	3	3
Altered sense of taste or smell	19	17	26	21	45	128	47.23	3.44	1.46	4	5
Heart palpitations	10	19	38	35	23	125	46.13	3.34	1.18	3	3

Joint pain	10	18	42	26	26	122	45.02	3.33	1.20	3	3
Dry cough	16	29	41	21	14	121	44.65	2.90	1.19	3	3
Chills or sweating	18	18	43	27	9	115	42.44	2.92	1.16	3	3
Other sleep symptoms	7	18	30	36	23	114	42.07	3.44	1.16	4	4
Disorientation or confusion	16	40	31	9	9	105	38.75	2.57	1.12	2	2
Loss of appetite	24	29	29	15	7	104	38.38	2.54	1.19	2	2[a]
Sore throat	31	29	26	9	5	100	36.90	2.28	1.15	2	1
Runny nose	33	31	16	10	7	97	35.79	2.25	1.23	2	1
Disturbed neurological sensations	13	16	32	24	10	95	35.06	3.02	1.19	3	3
Burning chest pain	17	18	25	19	13	92	33.95	2.92	1.31	3	3
Tachycardia	12	19	24	24	9	88	32.47	2.99	1.21	3	3[a]
Sneezing	23	33	20	7	5	88	32.47	2.30	1.12	2	2
Visual disturbances	17	27	22	6	10	82	30.26	2.57	1.25	2	2
Other eye symptoms	15	26	28	4	9	82	30.26	2.59	1.18	2.5	3
Bone pain	14	12	23	20	11	80	29.52	3.03	1.29	3	3
Cough (mucous)	24	15	20	12	6	77	28.41	2.49	1.29	2	1
Nerve pain	9	16	18	20	11	74	27.31	3.11	1.26	3	4
Muscle cramps	15	14	22	14	9	74	27.31	2.84	1.29	3	3
Nausea	21	22	16	6	9	74	27.31	2.46	1.32	2	2
Diarrhoea	19	24	17	8	5	73	26.94	2.40	1.19	2	2

Tremor	19	19	23	2	6	69	25.46	2.38	1.18	2	3
Rattling breathing	30	16	16	4	3	69	25.46	2.04	1.14	2	1
Slurred speech	16	27	14	4	5	66	24.35	2.32	1.14	2	2
Tinnitus	10	13	14	17	9	63	23.25	3.03	1.31	3	4
Fever	30	17	7	7	2	63	23.25	1.89	1.19	2	1
Stomach pain	19	15	19	5	4	62	22.88	2.35	1.19	2	1[a]
Elevated body temperature	32	10	11	3	5	61	22.51	2.00	1.29	1	1
Skin abnormalities or allergies	12	18	14	8	5	57	21.03	2.58	1.22	2	2
Heartburn/Reflux	15	16	17	6	2	56	20.66	2.36	1.10	2	3
Weight loss	18	13	14	3	7	55	20.30	2.42	1.34	2	1
Bladder control problems	19	13	8	10	4	54	19.93	2.39	1.34	2	1
Menstrual disorders	13	13	14	7	4	51	18.82	2.33	1.24	2	3
Skin rash	18	13	11	7	2	51	18.82	2.25	1.20	2	1
Constipation	18	11	16	3	1	49	18.08	2.14	1.06	2	1
Low body temperature	23	11	9	4	1	48	17.71	1.94	1.10	2	1
Vomiting	25	9	7	3	2	46	16.97	1.87	1.17	1	1
Hearing impairment	14	14	13	3	2	46	16.97	2.24	1.10	2	1[a]
Sleep apnea	16	12	8	4	5	45	16.61	2.33	1.35	2	1
Other temperature deviations	13	4	18	5	1	41	15.13	2.44	1.14	3	3
Protruding veins	15	4	12	6	1	38	14.02	2.32	1.23	2.5	1

Peeling skin	18	7	8	3	2	38	14.02	2.05	1.23	2	1
Hearing loss	14	13	7	2	2	38	14.02	2.08	1.12	2	1
New allergies	14	4	11	4	0	33	12.18	2.15	1.12	2	1
COVID toes	17	2	7	3	2	31	11.44	2.06	1.34	1	1
Hallucinations	20	6	2	2	0	30	11.07	1.53	0.90	1	1
Dermographism	15	2	6	3	3	29	10.70	2.21	1.45	1	1
Petechial rash	17	2	5	1	2	27	9.96	1.85	1.29	1	1
Bradycardia	12	10	3	1	0	26	9.59	1.73	0.83	2	1
Bloody cough	19	1	2	0	0	22	8.12	1.23	0.61	1	1
Anaphylactic reaction	14	1	1	1	2	19	7.01	1.74	1.41	1	1

[*] *Note.* [a] Multiple modes exist, the smallest value is shown
